# A Lady Doctor on Health and Disease

**Published:** 1887-05-21

**Authors:** Anna Longshore-Potts

**Affiliations:** at Prince's Hall


### A Lady Doctor on Health and Disease.

Mrs. Anna Longshore-Potts, M.D., at Prince's Hall.

London has just been visited by a female member of the American medical faculty, Mrs. Anna Longshore-Potts, M.D., of the Women's Medical College of Philadelphia. They manage these things differently in America. A sketch of one of Mrs. Potts' appearances may serve as a specimen of what is, perhaps fortunately, but little known on this side of the Atlantic. The lady, who has been on an extensive lecturing tour, made her *début* before a London audience some two years ago, and her platform lectures then excited a good deal of interest. Her second course of lectures, on "Health and Disease," will doubtless enhance her reputation in the particular department of science which she has made the special object of her study. Though her voice is somewhat harsh and her speech full of the American accent, her manner is singularly pleasant and attractive. To speak without cessation for two hours is by no means an easy matter, and the physical strain on many constitutions would, at the close, make itself manifest. Not so with Mrs. Potts. She appears to be as fresh at the close of her lecture as at the commencement. Her stores of information on every subject she discusses are apparently inexhaustible. As a popular medical lecturer Mrs. Potts has few, if any, equals. She never tires her audience with her subject. Dry facts about the wonderful mechanism of the human frame are, in her hands, endowed with a newness we had not dreamt of. Her lectures are full of quiet humour and trite witticisms, with here and there a neatly-turned bit of bathos.

It is not possible, within the compass of a single article, to go anything like exhaustively into the subjects dealt with by Mrs. Potts in her recent lectures at Prince's Hall. In the evening they were delivered to mixed audiences, while those in the afternoon were limited to ladies. On a large screen at the back of the platform a number of physiological diagrams were hung, and in front of these were placed the human skeleton and anatomical models. Speaking of the brain, Mrs. Potts combated the notion that nerves were intangible things, by showing that they were actual organs which might be taken up and dissected. The low, nervous state in which some persons found themselves was due to their not thinking about and cultivating their nerves. Mrs. Potts was grotesquely humorous in her description of persons with highly strung nerves, so sensitive that they could not bear the slamming of a door, nor even the rustling of a newspaper. Particular habits exhausted the nerve-force, especially the indulgence in narcotics, of which tobacco-smoking was one of the most common. In dealing with the subject of digestion, Mrs. Potts pointed out that all moving objects must change, that particles must be destroyed. There was a constant wear and tear of the body. Mental activity was more exhausting than physical exercise. To compensate for the loss which the body was always suffering, something must be added, and this was to be accomplished through the process of digestion. By the aid of the diagrams Mrs. Potts explained this function, tracing the passage of the food from its entry at the mouth through the esophagus into the stomach; the changes which it underwent there, the action of the small and the large intestines, the liver, pancreas, and glands connected with the same, and the special work accomplished by each.

There were, Mrs. Potts asserted, individuals who might not be able to answer correctly if the question were put to them, "Where is your mouth?" They would put their fingers to their lips, and there declare the mouth was; but this was not so. The lips were only folded doors, behind which was the mouth. It was this inside part which was so important to our comfort. The tongue was a most prominent part of the organ. Its surface was covered with a mucous membrane, on which were papillæ, like tufts of velvet. The tip of the tongue was the tasting part; the papillæ here were exceedingly sensitive. When we wanted to taste an article it was the tip of the tongue we used. The tongue was designed to move the food between the teeth, but it had another object—it was used in speech. In this respect it was to a great extent under the will, and we should be wise sometimes if we kept it still. Mrs. Potts not inaptly styled the epiglottis as a "little trap-door shutting up the opening to the lungs when we swallowed, and opening it again when we breathed." She next described the Eustachian tube—the passage from the throat to the internal ear—the channel leading upwards to the posterior nares or nostrils, and the uvula. Next she touched upon the teeth, the purpose of which was to masticate food, though the real masticators were the molars, which had been called the grindstones of the mouth, because they did the work. If a bone were fractured, it would grow again, but when the tooth was fractured it was "gone to stay"; till the doctor or the dentist put another there, nature would not. Cracking nuts would splinter the teeth, causing a cavity for decay to form. Some people never thought of washing their teeth any more than they did of washing their faces; but the teeth ought to be kept clean, free from tartar, a gritty substance which came from the saliva, and adhered to the teeth till it became an eighth, or even a quarter of an inch thick. However pretty the face, if the teeth were dark there would be no beauty anywhere: keep the teeth pearly white and the face well clean, and there would then be more beauty than all the artificial beauty one could apply. Every human being should have two things—a tooth-brush and a comb. The teeth were designed to chew the food, but through bad habits half the people lost them at twenty to twenty-five, and often at six and eight the teeth were going. A tooth, as soon as it began to decay, should be stopped. If we had our teeth examined by the dentist, we might keep them for a long, long time. When a tooth let the air in it would soon break down, for there would be corrosion going on. If we had it stopped, this process of decay would be prevented. In her experience gold-stopping was the cheapest in the end. If it did cost more we should consider what return we got. We should use our money for our health, our comfort, and our lives. She had some teeth that had been filled for twenty years; the dentist had her money, but she had her teeth, and could masticate with them and could not with her money. If the tooth was really broken down and decayed, the best thing was to have it taken away. Anæsthetics could be used, so there was no excuse in talking about pain.

In regard to taking food, Mrs. Potts contended that we took too much liquid, washing the food, so to speak, into the stomach. The horse did not do anything so silly, and why should we do worse than the horse? The liquids should come at times when there was no food in the mouth. She would not say, never have a liquid at the table. In dealing with the digestion of food in the stomach, Mrs. Potts gave a most amusing *résumé* of the famous experiments and examinations conducted by Dr. Beaumont on the soldier who had a portion of the wall of his stomach blown away. Alcohol she described as an irritant poison, and emphatically condemned its use as a beverage, illustrating her remarks of the action of alcohol on the coats of the stomach by diagrams of those (1) of a moderate drinker, and (2) of a drunkard. In powerful language and fervent voice the lecturer portrayed the agonising end to which over-indulgence in drink led men; the phantoms which the drunkard thought he saw in his delirium, and how he would scream, jump, and at last throw himself into eternity to save himself from what he fancied that he saw. She was ashamed, also, to acknowledge the amount of drunkenness among those of her own sex. Mrs. Potts touched on the feeding, etc., of infants, and her quiet satire on the ignorance of mothers evoked much applause. As to the best kind of food, she praised brown bread and porridge. The higher rose our civilisation the less meat we took; indeed, she believed the time would come when men would eat only fruits and vegetables, when there would be no killing of animals. We could not expect to stop all at once; but if we did eat meat, we should eat the clean kinds—those of animals that ate the grass and drank the running stream. Pigs she abominated, for they ate almost anything expect tobacco, and for that, and that alone, she respected them.

Mrs. Potts' concluding address was devoted to the blood, which she described as the foundation of the body, conveying oxygen to all its parts and carrying away its impurities. Rest was a most important factor in our existence. She spoke in favour of innovation. Harvey, who discovered the circulation of the blood, was an innovator, though he lived somewhat before his time. She begged her lady hearers to be innovators by giving up the wearing of corsets, and the gentlemen by abandoning their smoking. By way of a popular verdict, she asked all the gentlemen present who preferred a natural waist to a "small waist" to hold up their hands, the result being that not a single gentleman was sufficiently bold to advocate by vote the "small" or tight-laced waist.

Mrs. Potts has been accompanied on her tour by Dr. Charles Harrison, of the Chicago Medical College, who has lectured to men, his addresses drawing large audiences. Dr. Harrison will again lecture next month at Prince's Hall. Mrs. Longshore-Potts returns to America after an absence of five years.

